# Strong association of type 2 diabetes with degenerative lumbar spine disorders

**DOI:** 10.1038/s41598-021-95626-y

**Published:** 2021-08-13

**Authors:** Chul-Hyun Park, Kyoung-Bok Min, Jin-Young Min, Du Hwan Kim, Kyung Mook Seo, Don-Kyu Kim

**Affiliations:** 1 Department of Physical and Rehabilitation Medicine, Kangbuk Samsung Hospital, Sungkyunkwan University School of Medicine, Seoul, Korea; 2 Department of Preventive Medicine, College of Medicine, Seoul National University, Seoul, Korea; 3 Veterans Medical Research Institute, Veterans Health Service Medical Center, Seoul, Korea; 4 Department of Physical Medicine and Rehabilitation, College of Medicine, Chung-Ang University, Seoul, Korea

**Keywords:** Diabetes, Pain

## Abstract

Tantalizing connections between type 2 diabetes and degenerative lumbar spine disorders have become increasingly evident. However, the association of type 2 diabetes with degenerative lumbar spine disorders remains unclear. We sought to clarify the association between type 2 diabetes and lumbar spine disorders using nationwide data in Korea. Furthermore, we explored the association of diabetes with the prevalence of spinal procedures. The data in this study was obtained from Korean health claim database. Between 2016 and 2019, totals of 479,680 diabetes and 479,680 age- and sex-matched control subjects were enrolled. Patients with diabetes had more likely to have degenerative lumbar spine disorders and spinal procedures than controls. Using multivariate-adjusted analysis, patients with diabetes were at increased risk of being concomitantly affected by lumbar disc disorder [adjusted odds ratio 1.11 (95% confidence interval 1.10–1.12)], lumbar spondylotic radiculopathy [1.12 (1.11–1.13)], spondylolisthesis [1.05 (1.02–1.08)] and spinal stenosis [1.16 (1.15–1.18)], compared to controls. Furthermore, diabetic patients had an increased risk of undergoing lumbar spinal injection [1.13 (1.12–1.14)], laminectomy [1.19 (1.15–1.23)], and fusion surgery [1.35 (1.29–1.42)]. We demonstrated that type 2 diabetes was significantly associated with lumbar spine disorders and frequent spinal procedures. Our results suggest diabetes as a predisposing factor for lumbar spine disorders.

## Introduction

Low back pain is ranked first globally when it comes to attributing the cause of years lived with disability (YLDs). It is an increasingly major problem in an aging society^1^. Mechanical dysfunction accounts for up to 97% of low back pain diagnoses^2^. The major causes of mechanical lumbar spine disorders are degenerative intervertebral disc disorders, lumbar Spondylotic radiculopathy, spondylolisthesis and spinal stenosis, all part and parcel of a degenerative cascade of the lumbar spine^3,4^. Despite low back pain patients being highly prevalent the world over^1^, evidence of risk factors for the degenerative lumbar spine disorders remains lacking. For many years, systemic reviews and meta-analysis pointed to genetic factors, aging, obesity, smoking and occupational factors being the risk factors for low back pain^5–9^. However, it has been noted that degeneration of lumbar spine disorders can be accelerated by metabolic factors and a toxic environment^10^. Therefore, the associations of lumbar spine disorders with metabolic diseases such as diabetes mellitus needs to be established^11–13^.

Type 2 diabetes can lead to various complications involving major organs, and gives rise to degenerative changes of the organs^14^. Various pathological changes in spine structure such as loss of disc height, decreased vertebral bone mass, and endplate sclerosis are well documented in diabetic model which reflect similar degenerative findings in human intervertebral discs^15–17^. In clinical data, there is conflicting evidence with regards to a relationship between diabetes and lumbar spine disorders. A close relation between type 2 diabetes and low back pain was reported^18–20^. However, a few studies reported that type 2 diabetes was linked to degenerative lumbar spine disorders^21,22^. A recent twin cohort study found that there was no significant association between diabetes and lumbar disc disorder^23^. Therefore, it is necessary to confirm the associations between diabetes and degenerative lumbar spine disorders by a large-scaled, population-based study. Furthermore, to determine the impact of diabetes on the outcome of lumbar spine disorders, the prevalence of spinal procedures in diabetes patients should be investigated. To the best of our knowledge, a population-based study of the associations of diabetes with degenerative lumbar spine disorders and the prevalence of undergoing spinal procedures has not yet been studied.

Therefore, this study investigated the association between type 2 diabetes mellitus and degenerative lumbar spine disorders using a nationwide, population-based data in Korea. Furthermore, we explored the impact of diabetes on the prevalence of lumbar spinal injection and surgical procedures.

## Results

### Baseline characteristics

**Figure 1:**
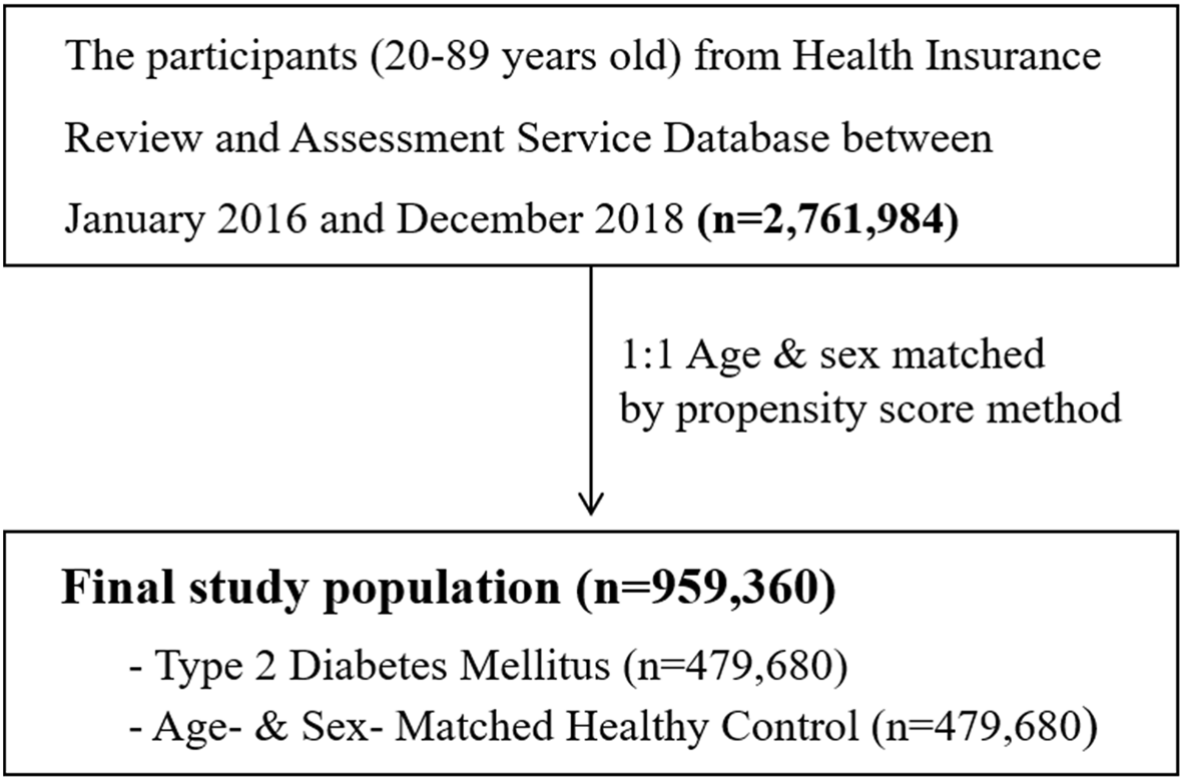
Selection of the study population.

Table 1 shows the baseline characteristics of patients with type 2 diabetes in unmatched and matched population-based controls. The study ultimately enrolled 479,680 subjects each, both for patients with diabetes as well as for age- and sex- matched control subjects. Thus, a final study population were 959,360 subjects (Fig. 1). In each group, 240,759 subjects (50.2%) were male (*p* = 1.0). For the type of beneficiary and underlying diseases, the proportions of medical aid, hypertension, chronic kidney disease, and dyslipidemia were significantly higher in the diabetes group than in matched control group, respectively (all *p* value < 0.0001).

**Table 1:** Baseline characteristics of patients with type 2 diabetes in unmatched and matched population-based controls.

Baseline characteristics	Unmatched subjects		*p* value	Matched subjects		*p* value
	Control (n = 2,282,304)	Diabetic patients (n = 479,680)		Control (n = 479,680)	Diabetic patients (n = 479,680)	
**Age (year)**						
20–29	314,477 (13.8)	3,273 (0.7)	< 0.0001	3,273 (0.7)	3,273 (0.7)	1.0
30–39	410,357 (18.0)	12,965 (2.7)		12,965 (2.7)	12,965 (2.7)	
40–49	416,885 (18.3)	40,290 (8.4)		40,290 (8.4)	40,290 (8.4)	
50–59	464,584 (20.4)	101,035 (21.1)		101,035 (21.1)	101,035 (21.1)	
60–69	315,449 (13.8)	128,728 (26.8)		128,728 (26.8)	128,728 (26.8)	
≥ 70	360,552 (15.7)	193,389 (40.3)		193,389 (40.3)	193,389 (40.3)	
**Sex**						
Male	997,275 (43.7)	240,759 (50.2)	< 0.0001	240,759 (50.2)	240,759 (50.2)	1.0
Female	1,285,029 (56.3)	238,921 (49.8)		238,921 (49.8)	238,921 (49.8)	
**Type of beneficiary**						
Medical insurance	2,171,213 (95.1)	425,153 (88.6)	< 0.0001	444,842 (92.7)	425,153 (88.6)	< 0.0001
Medical aid	111,091 (4.9)	54,527 (11.4)		34,838 (7.3)	54,527 (11.4)	
**Hypertension**						
Yes	604,615 (73.5)	345,527 (73.9)	< 0.0001	227,737 (47.5)	345,527 (73.9)	< 0.0001
No	1,677,689 (26.5)	125,153 (26.1)		251,943 (42.5)	125,153 (26.1)	
**Chronic kidney disease**						
Yes	23,984 (98.9)	38,839 (8.0)	< 0.0001	9,547 (2.0)	38,839 (8.0)	< 0.0001
No	2,258,320 (1.1)	440,841 (92.0)		470,133 (98.0)	440,841 (92.0)	
**Dyslipidemia**						
Yes	144,498 (6.3)	98,099 (20.5)	< 0.0001	47,260 (9.9)	98,099 (20.5)	< 0.0001
No	2,137,806 (93.7)	381,581 (79.5)		432,420 (90.1)	381,581 (79.5)	

Data are presented as number (percentage, %).

### Prevalence of lumbar spine disorders and spinal procedures

**Figure 2:**
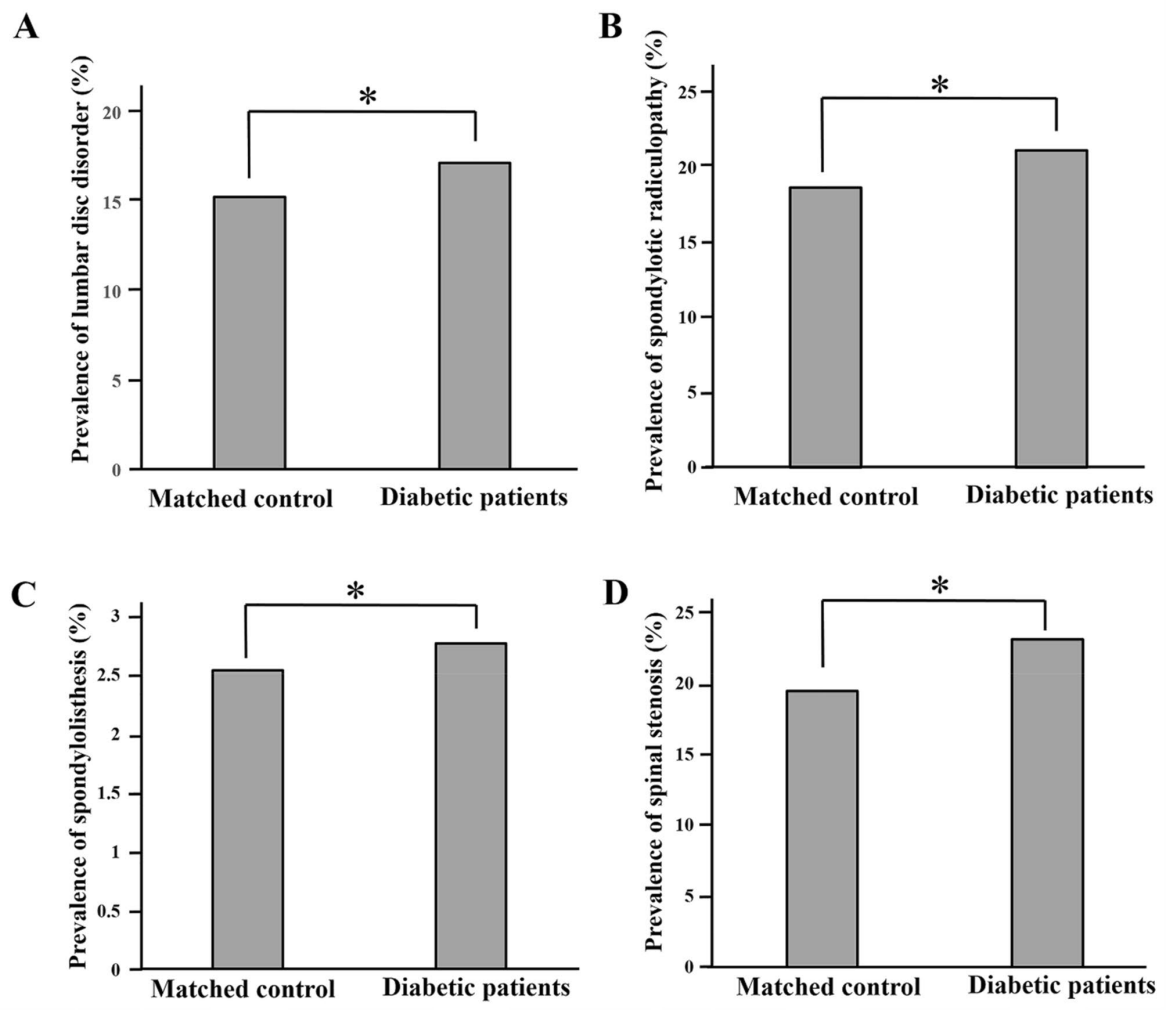
Prevalence of (**A**) lumbar disc disorder, (**B**) spondylotic radiculopathy, (**C**) spondylolisthesis, (**D**) spinal stenosis in matched control and diabetic patients. *p < 0.0001.

In diabetic patients, the prevalence of lumbar disc disorder and spondylotic radiculopathy were 17.2% and 21.2%, respectively, showing markedly higher compared to the control group (*p* < 0.0001; Fig. 2). The diabetes group also exhibited a higher prevalence in lumbar spondylolisthesis (2.8%), and spinal stenosis (23.1%) than the control group, respectively (*p* < 0.0001).

**Figure 3:**
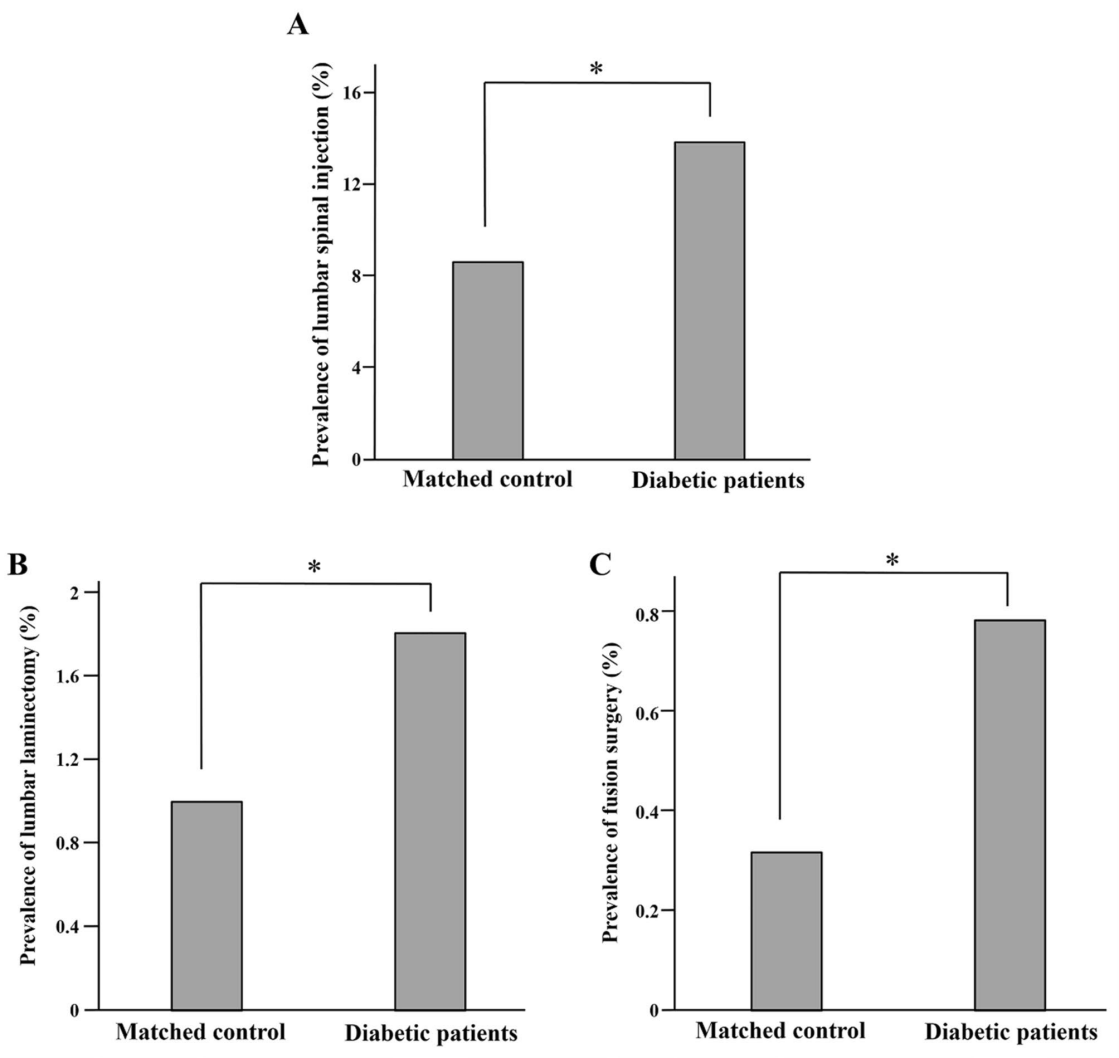
Prevalence of (**A**) lumbar spinal injection, (**B**) lumbar laminectomy, and (**C**) fusion operation in matched control and diabetic patients. *p < 0.0001.

The prevalence of lumbar spinal injection was significantly higher in the diabetes group than in the control group (13.9% vs. 8.6%; *p* < 0.0001) (Fig. 3). Furthermore, the diabetes group had more lumbar laminectomy (1.8% vs. 1.0%; *p* < 0.0001), and lumbar fusion surgery (0.8% vs. 0.3%; *p* < 0.0001) than those of the control group, respectively.

### Association of diabetes mellitus with lumbar spine disorders and spinal procedures

Patients with diabetes were at an increased risk of comorbid lumbar disc disorder (aOR 1.11, 95% CI 1.10–1.12) and lumbar spondylotic radiculopathy (aOR 1.12, 95% CI 1.11–1.13) compared with the control group, even after adjustments for hypertension, chronic kidney disease, dyslipidemia, and the type of beneficiary (Table 2). Furthermore, diabetic patients had a significantly higher risk of lumbar spondylolisthesis (aOR 1.05, 95% CI 1.02–1.08) and spinal stenosis (aOR 1.16, 95% CI 1.15–1.18), respectively.

**Table 2:** Association of type 2 diabetes with lumbar disc disorder, spondylotic radiculopathy spondylolisthesis, and spinal stenosis.

Outcome	Groups	Crude OR (95% CI)	Adjusted* OR (95% CI)
Lumbar disc disorder	Matched control	Reference	Reference
	Diabetic patients	1.15 (1.14–1.17)	1.11 (1.10–1.12)
	*p* value	< 0.0001	< 0.0001
Spondylotic radiculopathy	Matched control	Reference	Reference
	Diabetic patients	1.17 (1.16–1.18)	1.12 (1.11–1.13)
	*p* value	< 0.0001	< 0.0001
Spondylolisthesis	Matched control	Reference	Reference
	Diabetic patients	1.10 (1.06–1.12)	1.05 (1.02–1.08)
	*p* value	< 0.0001	< 0.0001
Spinal stenosis	Matched control	Reference	Reference
	Diabetic patients	1.24 (1.23–1.26)	1.16 (1.15–1.18)
	*p* value	< 0.0001	< 0.0001

*OR* Odds ratio, *CI* Confidence interval.

*Adjusted by age, sex, type of beneficiary, hypertension, chronic kidney disease, and dyslipidemia.

Diabetic patients were at increased risk of undergoing lumbar spinal injection (aOR 1.13, 95% CI 1.12–1.14), lumbar laminectomy (aOR 1.19, 95% CI 1.15–1.23), and fusion surgery (aOR 1.35, 95% CI 1.29–1.42) compared with the controls, respectively (Table 3).

**Table 3:** Association of type 2 diabetes with the prevalence of lumbar spinal injection, laminectomy, and lumbar fusion.

Outcome	Groups	Crude OR (95% CI)	Adjusted* OR (95% CI)
Spinal injection	Matched control	Reference	Reference
	Diabetic patients	1.19 (1.17–1.20)	1.13 (1.12–1.14)
	*p* value	< 0.0001	< 0.0001
Lumbar laminectomy	Matched control	Reference	Reference
	Diabetic patients	1.26 (1.22–1.30)	1.19 (1.15–1.23)
	*p* value	< 0.0001	< 0.0001
Lumbar fusion	Matched control	Reference	Reference
	Diabetic patients	1.48 (1.40–1.56)	1.35 (1.29–1.42)
	*p* value	< 0.0001	< 0.0001

*OR* Odds ratio, *CI* Confidence interval.

*Adjusted by age, sex, type of beneficiary, hypertension, chronic kidney disease, and dyslipidemia.

### Subgroup analysis according to diabetic complication

**Figure 4:**
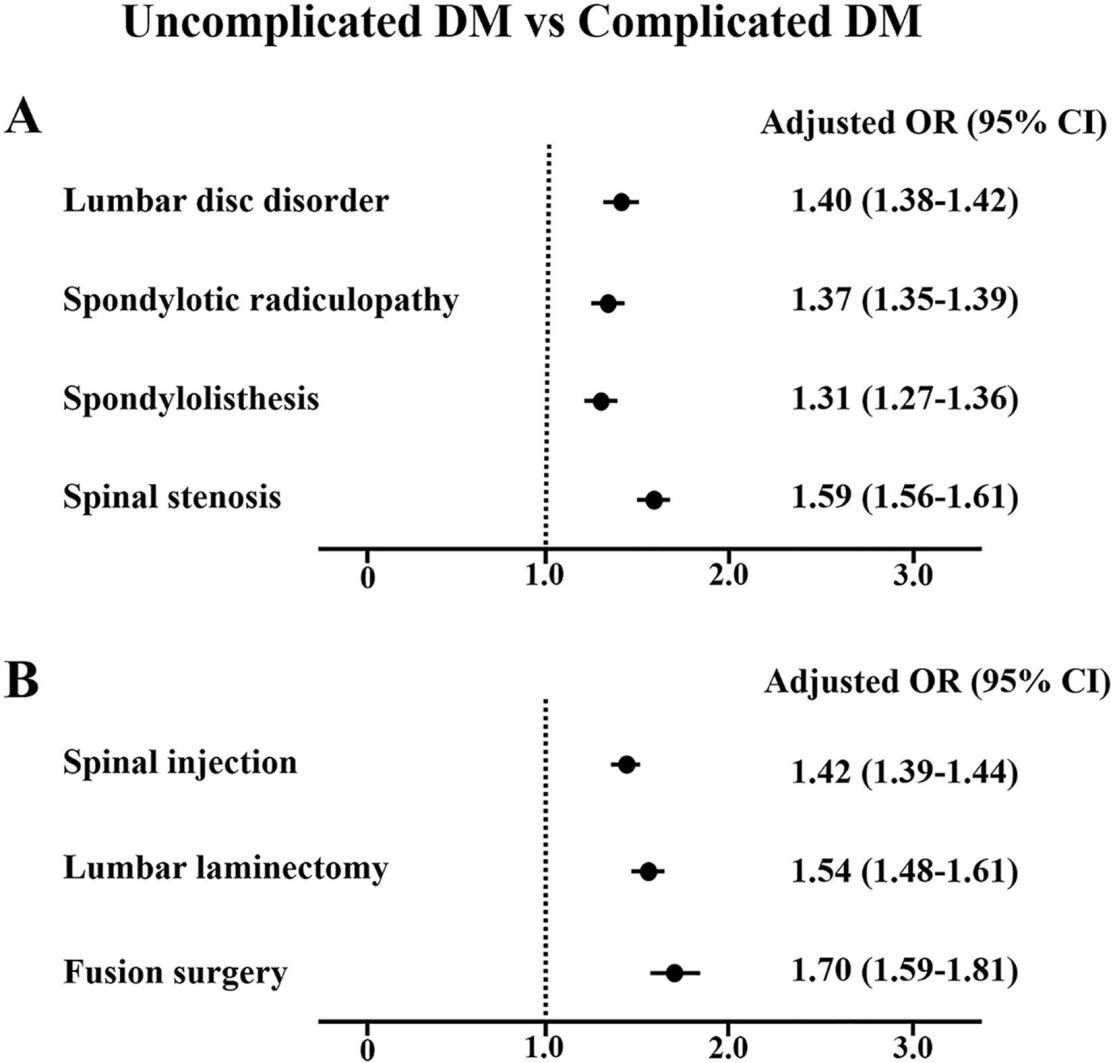
Association of complicated diabetes with (**A**) lumbar spine disorders and (**B**) spinal procedures compared with uncomplicated diabetes. Adjusted OR (95% CI) was estimated by multiple logistic regression analysis with adjustments for hypertension, chronic kidney disease, dyslipidemia, and the type of beneficiary. DM, diabetes mellitus; OR, odds ratio; CI, confidence interval.

In subgroup analysis of diabetic complication, patients with complicated diabetes had a significantly higher risk of lumbar disc disorder (aOR 1.40, 95% CI 1.38–1.42) and spondylotic radiculopathy (aOR 1.37, 95% CI 1.35–1.39) compared with uncomplicated diabetes (Fig. 4A). Complicated diabetes had an increased risk of lumbar spondylolisthesis (aOR 1.31, 95% CI 1.27–1.36) and spinal stenosis (aOR 1.59, 95% CI 1.56–1.61), respectively. Complicated diabetes was strongly associated with undergoing lumbar spinal injection (aOR 1.42, 95% CI 1.39–1.44), lumbar laminectomy (aOR 1.54, 95% CI 1.48–1.61), and fusion surgery (aOR 1.70, 95% CI 1.59–1.81), respectively (Fig. 4B).

The associations of individual diabetic complication with lumbar spine disorders and spinal procedures were evaluated as referenced to uncomplicated diabetes (Supplementary Figs. S1–S4). Diabetic neuropathy was at highest risk for lumbar spinal disorders and spinal procedures. Retinopathy and arthropathy were highly associated with lumbar spine disorders and spinal injection. However, nephropathy was negatively associated with lumbar spine disorders and procedures.

## Discussion

The present study revealed that type 2 diabetes was strongly associated with lumbar spine disorders including lumbar disc disorder, lumbar spondylotic radiculopathy, spondylolisthesis, and spinal stenosis. Furthermore, patients with diabetes exhibited higher risks of undergoing lumbar injections and surgical procedures, suggesting diabetes is closely related to aggravating lumbar spine conditions, which ultimately necessitates more spinal procedures. Among diabetic patients, diabetic complication was presented as a significant risk factor for lumbar spine disorders and frequent spinal intervention. To the best of our knowledge, this is the first nationwide epidemiological evidence of associations of type 2 diabetes with major lumbar spine disorders and spinal procedures. This study had the following strengths: large sample size with matched control subjects, reliable disease diagnosis purely by physicians, and minimized effect of socioeconomic status under the National Health Insurance system.

### Association of type 2 diabetes mellitus and degenerative lumbar spine disorders

Close relationship between diabetes and lumbar spine disorders has long been recognized in animal studies. Diabetic models showed several deleterious changes such as increasing toxic end products of glycation (advance glycation end products, AGEs), expression of matrix metalloproteinases (MMP)-2 related to degradation of extracellular matrix, and hyperglycemia-induced inflammation in intervertebral disc, which promotes intervertebral disc degeneration process^15–17,24–26^. However, despite the mechanisms for diabetes-related pathway demonstrated in animal studies, clinical evidence for the relationship between diabetes and intervertebral disc degeneration remains still lacking^27^. There are some clinical studies that chronic back pain was associated with type 2 diabetes, findings in line with our study^19,20,28^. Eivazi et al. demonstrated that the prevalence of chronic low back pain were 63.4% in diabetic adults and 47.0% in non-diabetic adults^19^. With respect to degenerative spine disorders, Agius et al. presented that the lower height of lumbar discs was significantly associated with type 2 diabetes, suggesting that degenerative disc disorders is closely related to diabetes^22^. In contrast, Fabiane et al. reported that there was no significant association between diabetes and lumbar disc disorder in twin volunteers^23^. One recent study has raised a possible connection between diabetes and spinal stenosis, revealing a high frequency of diabetes in patients with spinal stenosis^29^. Previous clinical studies for relation between diabetes and lumbar spine disorders have included only a small number of patients and was inconclusive. Therefore, this study being a large-population study is important in which it confirms the relationship between type 2 diabetes and the major lumbar spine disorders such as lumbar disc disorder, spondylotic radiculopathy, spondylolisthesis, and spinal stenosis.

### Diabetes associated with increased prevalence of lumbar spinal procedures

We firstly report the impact of diabetes on the prevalence of lumbar spine procedures, suggesting diabetes be recognized as a possible contributing factor of aggravating lumbar spine disorders. This study presented that diabetic patients underwent more lumbar surgeries than those without diabetes. Furthermore, diabetes was suggested as a potential risk factor for increasing lumbar surgery rates including lumbar laminectomy with/without discectomy, and fusion surgery. The indications for these lumbar surgeries were herniated lumbar disc disorder with neurologic signs of lumbar laminectomy with/without discectomy, and spinal instability, advanced spondylolisthesis, or spinal stenosis for lumbar fusion operation. Because these indications are the most critical value in clinical field for patients with degenerative lumbar spine disorder, we focused on these lumbar surgeries in consideration of those patients having a severe surgery-indicated status of their lumbar spine. Therefore, the present finding that diabetes was significantly associated with increased lumbar spine surgeries is of critical importance, since this suggests that diabetes can be a predisposing factor for increasing the severity of lumbar spine disorders, leading ultimately to the necessity of a surgical procedure.

### Association of diabetic complication with lumbar spine condition

We showed that diabetic complication significantly associated with increased prevalence of lumbar spine disorders and spinal procedures compared with uncomplicated diabetes. Although these complications might exist at the time of the clinical diagnosis, the prevalence of diabetic complications increases over time. Furthermore, uncontrolled hyperglycemia may accelerate the progression of these complications. Thus, the rationale for regular health-checkups is that the frequency of the diabetic complications, especially vascular complications, increases with disease duration^30^. Thus, it is possible to assume that the status of diabetic patients with complications may be categorized as long-standing or uncontrolled diabetes^14,31^. In a recent study^21^, a longer duration of type 2 diabetes was suggested to be a risk factor for lumbar disc degeneration, as shown by a cross-sectional study using magnetic resonance imaging (MRI), a result which is in line with our findings. Because diabetic complication is highly related to hyperglycemia and long-term suffering with diabetes^32,33^, this could produce toxic byproducts and inflammatory cytokines which could give harmful consequences for lumbar spine. Thus, long-term suffering from diabetes can aggravate lumbar spine condition, which possibly ends up necessitating lumbar spine procedures. Therefore, the present findings of the close relationship between complicated diabetes and lumbar spine disorders suggest physicians should take monitoring glycemic control more into consideration when treating back pain patients with a history of diabetes.

### Which diabetic complications are highly associated with lumbar spine disorders and procedures?

Multiple diabetic complications were included in this study to find out which complication is most associated with lumbar spine disorders and spine procedures. Of the many diabetes related complications, microvascular complications such as retinopathy, neuropathy, and nephropathy were included because these complications are of importance for the prognosis of diabetes^23,33^. Furthermore, diabetic arthropathy, being closely related to diabetic neuropathy was added to the analysis. Of these complications, diabetic neuropathy and arthropathy were the most highly associated lumbar spine disorders. These forms of diabetes, either neuropathy or arthropathy, had a high risk of more spine intervention and surgeries. Although arthropathy has multifactorial mechanical and vascular causes, arthropathy is known to be caused by deficit in proprioception secondary to diabetic neuropathy^34^. It is also called as neuropathic arthropathy or Charcot arthropathy. Therefore, diabetic neuropathy would be a key contributing factor associated with aggravating lumbar spine disorders. There are a several possible explanations. First, diabetic neuropathy increases the susceptibility of nerves to ischemic changes^35,36^. These susceptible nerves can be trunks, roots, or peripheral nerves. One of important pathogenesis of radicular pain secondary to herniated lumbar disc disorders is mechanical compression of nerve roots and chemical irritations, thus having intra-neural edema formation and ischemic insults nerve roots^35,37^. Therefore, under conditions of diabetic neuropathy, the damage in the nerve root could be accelerated, possibly becoming more severe radicular pain in a herniated lumbar disc disorder. Second, mechanical sensitivity can be increased in afferent fibers of diabetic nerves^38–40^. Because diabetes nerves are known to have damaged myelinated and unmyelinated afferents, spontaneous aberrant activities from nociceptive C-fibers in diabetic nerves has been reported^40^. These abnormal hyperactivities of nerves are susceptible to mechanical stimulation^38,39^. Approximately 97% of low back pain is thought to be mechanical origin pain^2^. Therefore, the hypersensitive state of diabetic nerve roots evoked by mechanical stimulation could be highly associated with painful lumbar spine disorders, thus having more spine interventions and surgeries in diabetic neuropathy.

The present study showed that diabetic retinopathy was a significant complication associated with increasing prevalence of lumbar spine disorders, though not as strongly as diabetic neuropathy or arthropathy. On the other hand, diabetic nephropathy was found not to be associated with increasing prevalence of lumbar spine disorders. Diabetic nephropathy is a well-known late-onset complication of diabetes, which would tend to imply a positive correlation with lumbar spine disorders. There are a several explanations for why this is not the case. First, although retinopathy, neuropathy, and nephropathy are microvascular complications, the relationship among the complications can be different. Recent systemic review and meta-analysis^41^ which presented the associations between the diabetic complications, showed that retinopathy was significantly associated with nephropathy and neuropathy, respectively. However, nephropathy had no association with neuropathy. Thus, even while neuropathy was shown to be closely related to lumbar spine disorders in this study, it may well be that nephropathy possesses different pathways, when compared to neuropathy. Second, the subjects of this study were sampled largely from inpatient populations. Of diabetic complications, nephropathy is a very long-standing and advanced complication. Thus, subjects with a history of admission, who were included in this study, may be admitted due to their serious medical problems. These patients’ lumbar spine condition may not be considered due to an inability to undergo spine interventions and surgical procedures on them. Given this, lumbar spine disorders would be under-diagnosed in patients with diabetic nephropathy.

### Limitations

There are a few limitations. First, we used national insurance claim data which can have fail to include prescriptions outside of insurance coverage (though this is less than 1% in South Korea), included possible incorrect diagnosis codes, and misclassification errors. But, due to the large-sample population, we believe such errors had little effect on our results. The major strength of this study is that it is a representative result drawn from a nationwide database of the South Korean population. Thus, this minimizes recruitment bias, or selection bias, something which is unavoidable in a cohort study. Second, although we have demonstrated that diabetic neuropathy was associated with lumbar spine disorders, clinical differentiation of lumbar radiculopathy from the diabetic radiculopathy is difficult due to similar clinical presentations between the diseases. Thus, the over-estimation of the association between neuropathy and lumbar spine disorders might be concerned. Nevertheless, the two diseases can be differentiated by electromyography or some different clinical features^42^. Because the diagnosis of the diseases was solely dependent on physician decision in this study, the misclassification errors are expected to be minimized. Third, there can be a selection bias. Patients with diabetes would be more likely to visit the hospital than those without diabetes. It is possible that screening for lumbar spine disorder could be conducted earlier in diabetic patients. Lastly, this study is a retrospective, cross-sectional study that cannot confirm the causal relationship. Therefore, a future study is still needed to find out the predictive effect of diabetes on degenerative lumbar spine disorders. Furthermore, we should investigate whether poor glycemic control in patients with diabetes would affect the lumbar spine condition in a prospective cohort study.

## Conclusions

In conclusion, this current, nationwide population-based study demonstrated that type 2 diabetes is associated with increased risks of comorbid lumbar spine disorders and undergoing spinal procedures. Furthermore, patients with complicated diabetes had greater risks for lumbar spine disorders and frequent spinal procedures, which confirms the association between type 2 diabetes and lumbar spinal diseases. This work suggests that diabetes may be a predisposing factor for degenerative lumbar spine disorder, which highlights the importance of early detection for lumbar spine pathology in diabetic patients. Future long-term prospective studies are warranted to investigate the underlying pathophysiological mechanism.

## Materials and methods

### Data source and study subjects

We conducted a 3-year population-based, matched case–control study using the 2016 to 2018 claims database of the Health Insurance Review and Assessment Service-National Inpatient Sample (HIRA-NIS). The Korean National Health Insurance Service is a single payer social insurance system with compulsory enrollment, in which the raw claims dataset of the HIRA includes almost all Korean citizens, approximately 46 million population^43^. HIRA-NIS is a population-based representative annual sample of one million patients’ health data covering the entirety of all regions of South Korea^43^. This database utilizes a probabilistic weighted sample extraction method (sampling rate: 13% of total inpatients population, 1% of total out-patients population), which can be converted to detail the entire Korean population. All clinics and hospitals in Korea must provide the HIRA with information on diagnosis, treatment, and surgery.

The HIRA claims for inpatient and outpatient diagnoses, treatments and surgical procedures are coded using the International Statistical Classification of Diseases and Related Health Problems, 10th revision (ICD-10), and the Korean Drug and Anatomical Therapeutic Chemical Codes^44,45^. The HIRA regularly audits the database, which have been used in a variety of peer-review publications. The data derived from the database is thus considered reliable^43,46^.

Based on a definition of diabetes from a previous study^45^, patients with type 2 diabetes were defined as showing the presence of identical E11–E14 (ICD-10) codes at least two times, or an E11–E14 code and the prescription of anti-diabetic medications, which entails the use of sulfonylurea, meglitinides, biguanides, thiazolidinediones, dipeptidyl peptidase-4 inhibitors, a-glucosidase inhibitors, sodium-glucose co-transporter 2 inhibitors, glucagon-like peptide 1 agonists, or insulin. Furthermore, the data was searched again to identify patients who had complications related to diabetes for a subgroup analysis. These subjects were included if they had any of the following diagnoses of diabetic complications: diabetic retinopathy (diagnosis code: H360), diabetic nephropathy (N083, E112, E122, E132, or E142), diabetic arthropathy (M142 or M146), and diabetic neuropathy (G590, G632, or G990)^47,48^.

Among the participants aged 20–89 years from HIRA database between January 2016 and December 2018 (n = 2,761,984), patients with type 2 diabetes were selected as the definition (Fig. 1). The prevalence of type 2 diabetes was 17.4%. For the control group, subjects were enrolled by using age- and sex- matched sampling methods that incorporate the propensity score at a 1:1 ratio of controls to diabetic patients.

### Outcome measures

The disorders of interest were degenerative lumbar spine diseases, which are major causes of mechanical low back pain. Based on the previous studies^49,50^, the HIRA national database was searched to identify patients who had a primary diagnosis of (i) degenerative lumbar disc disorder (diagnosis code: M51, M512, M513, M518, M519), (ii) degenerative or spondylotic lumbar radiculopathy (M472, M511, or M541), (iii) degenerative lumbar spondylolisthesis (M431), or (iv) degenerative lumbar spinal stenosis (M48 or M995) were identified. These subjects were selected if they had undergone any of the spinal injections including lumbar epidural steroid injection (procedure code: LA322, HA102), selective nerve root injection (LA352-LA357), and facet or perifacet joint injection (LA358-LA359). Furthermore, the subjects were selected if they had any of the following primary spinal surgeries: (i) lumbar laminectomy with or without discectomy (procedure code: N1493, N14930, N1499, or N2499), and (ii) lumbar fusion operation (N2470, N1460, N1466, N1469, N0466, or N0469).

Confounding factors used in this study included type of beneficiary, hypertension (I10-I13), chronic kidney disease (N18), and dyslipidemia (E78).

### Indications of lumbar spine surgeries

Surgical indications of degenerative lumbar disc disorder, spondylotic radiculopathy, spondylolisthesis, and spinal stenosis are noted by the National Health Insurance Corporation (NHIC) of the South Korea^50,51^. Almost all clinics and hospitals in South Korea have adopted the recording requirements of lumbar spine surgery, notifying the NHIC for financial reimbursement. The standard of surgical treatment for subjects with lumbar disc disorder and spondylotic radiculopathy is lumbar laminectomy, with or without discectomy, in patients with neurologic sign (e.g. numbness, paresthesia, or weakness), or chronic intractable pain despite having received conservative treatment for a period of time exceeding 3 months. For lumbar spondylolisthesis and spinal stenosis, a lumbar fusion operation is indicated, if lumbar spine instability or foraminal stenosis is present. Therefore, these requirements of the Korean NHIC are regarded as surgical indications for patients.

### Statistical analysis

Prevalence was estimated as the number of cases divided by the study population. To compare the prevalence of outcomes between the diabetes and matched control groups, chi-square test was used. To determine the association of type 2 diabetes with the lumbar spine disorders and spinal procedures, a multiple logistic regression analysis was performed with adjustments for hypertension, chronic kidney disease, dyslipidemia, and the type of beneficiary. Adjusted odds ratios (aOR) were estimated to determine the association of diabetes with lumbar spine disorders and spinal procedures compared with the matched control group. Furthermore, a subgroup analysis was performed by the presence of diabetic complication. All statistical analyses, including analyses of the matching data, were performed using SAS software (SAS Institute, Inc., Cary, North Carolina). A *p* value below 0.05 was considered statistically significant.

### Ethics approval

Ethics approval for the study protocol and data analysis was obtained from the Institutional Review Board (IRB) of Seoul National University Hospital, Seoul, Republic of Korea (IRB number: E-2001-001-1090). This study was conducted in accordance with the 1975 Declaration of Helsinki. Informed consent was waived by the Institutional Review Board of Seoul National University Hospital because the researchers retrospectively assessed de-identified data for analytical purposes.

## Supplementary Information

Supplementary Information.

## Data Availability

The datasets analyzed for the study are available from the corresponding author on reasonable request.
