# Supplementary material for: Strong association of type 2 diabetes with degenerative lumbar spine disorders
Source: Sci Rep. 2021 Aug 13;11:16472. doi: 10.1038/s41598-021-95626-y (PMC8363740; doi:10.1038/s41598-021-95626-y)
Supplement: Supplementary file 1 — Supplementary Information. [file 41598_2021_95626_MOESM1_ESM.docx]

**Supplementary information**

**Strong association of type 2 diabetes with degenerative lumbar spine disorders**

Chul-Hyun Park, Kyung-Bok Min, Jin-Young Min, Kyung Mook Seo, Du Hwan Kim, Don-Kyu Kim

**Table of Contents**

**Supplementary Figure S1.** Association of Diabetic Retinopathy with (A) Lumbar Spine Disorders and (B) Spinal Procedures.

**Supplementary Figure S2.** Association of Diabetic Neuropathy with (A) Lumbar Spine Disorders and (B) Spinal Procedures.

**Supplementary Figure S3.** Association of Diabetic Nephropathy with (A) Lumbar Spine Disorders and (B) Spinal Procedures.

**Supplementary Figure S4.** Association of Diabetic Arthropathy with (A) Lumbar spine Disorders and (B) Spinal Procedures.


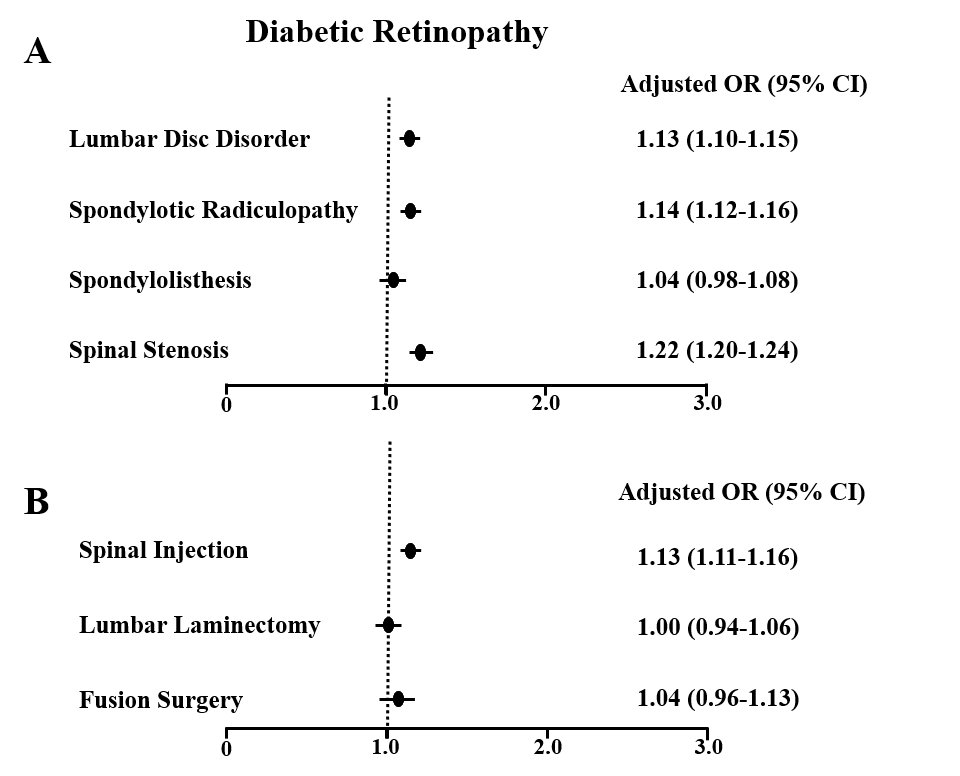


**Supplementary Figure S1.** Association of Diabetic Retinopathy with (A) Lumbar Spine Disorders and (B) Spinal Procedures.

Adjusted OR (95% CI) was estimated by multiple logistic regression analysis with adjustments for hypertension, chronic kidney disease, dyslipidemia, and the type of beneficiary. DM, diabetes mellitus; OR, odds ratio; CI, confidence interval.


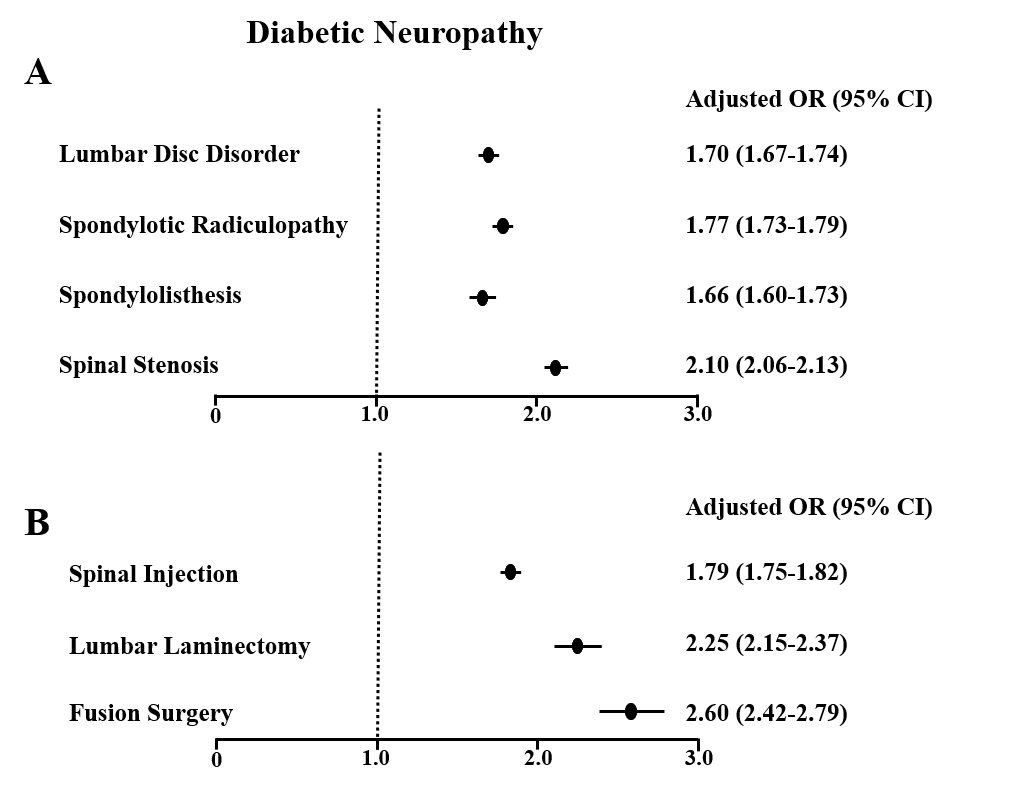


**Supplementary Figure S2.** Association of Diabetic Neuropathy with (A) Lumbar Spine Disorders and (B) Spinal Procedures.

Adjusted OR (95% CI) was estimated by multiple logistic regression analysis with adjustments for hypertension, chronic kidney disease, dyslipidemia, and the type of beneficiary. DM, diabetes mellitus; OR, odds ratio; CI, confidence interval.


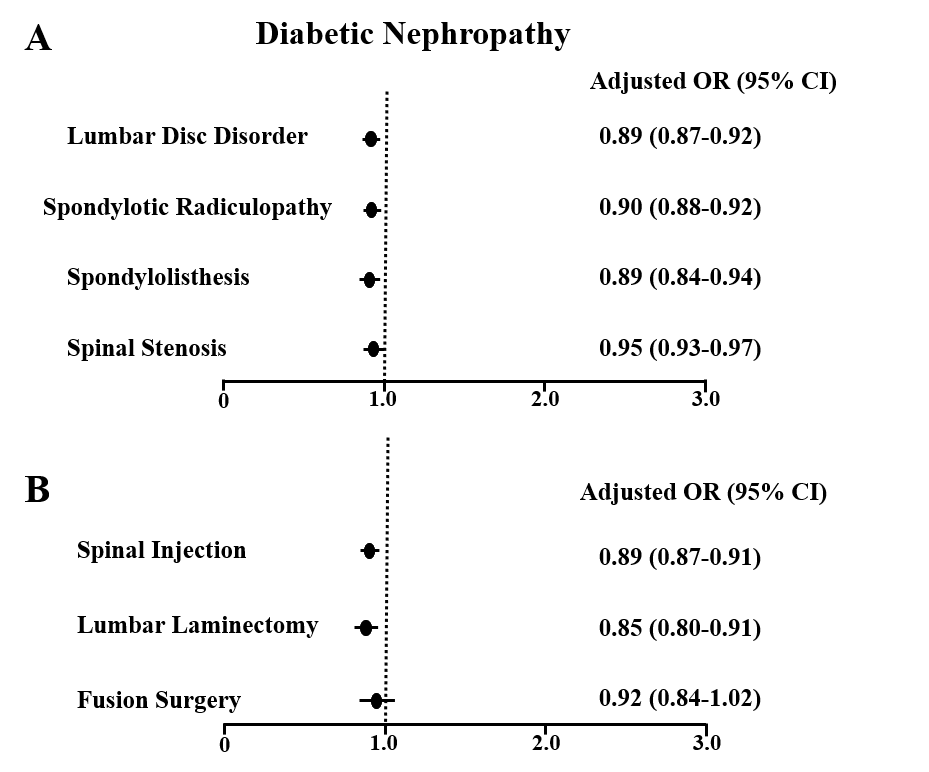


**Supplementary Figure S3.** Association of Diabetic Nephropathy with (A) Lumbar Spine Disorders and (B) Spinal Procedures.

Adjusted OR (95% CI) was estimated by multiple logistic regression analysis with adjustments for hypertension, chronic kidney disease, dyslipidemia, and the type of beneficiary. DM, diabetes mellitus; OR, odds ratio; CI, confidence interval.


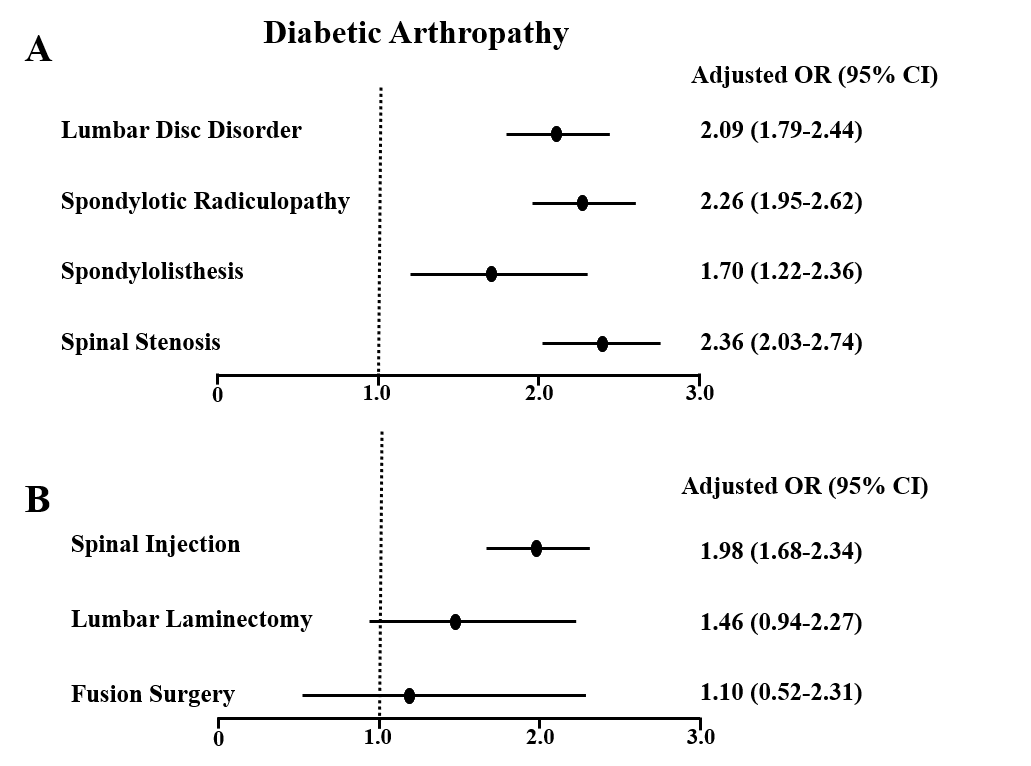


**Supplementary Figure S4.** Association of diabetic arthropathy with (A) Lumbar spine disorders and (B) Spinal procedures.

Adjusted OR (95% CI) was estimated by multiple logistic regression analysis with adjustments for hypertension, chronic kidney disease, dyslipidemia, and the type of beneficiary. DM, diabetes mellitus; OR, odds ratio; CI, confidence interval.
